# Accuracy of recording and reporting of malaria rapid diagnostic tests in Nigeria

**DOI:** 10.1186/s12936-025-05601-5

**Published:** 2025-11-07

**Authors:** Sunday Atobatele, Sidney Sampson, Evelyn Orya, Onyebuchi Okoro, Ese Akpiroroh, Hilary Okagbue, Shiva D. Gab-deedam, Olufisayo Bademosi, Ginini Atu, Kim A. Lindblade, Al-Mustapha Mukhtar, Oluwafisayo Ayodeji, Eugene C. Eugene, John J. Aponte, Natalie Galles, Michael Humes, Kevin Griffith, Shawna Cooper, Nnenna Ogbulafor, Chukwu Okoronkwo, Godwin Ntadom

**Affiliations:** 1 Sydani Group, Abuja, Nigeria; 2 Sydani Institute for Research and Innovation, Plot 1422 Independence Avenue, Central Business District, Abuja, Nigeria; 3 National Malaria Elimination Programme, Abuja, Nigeria; 4 PMI Insights Project, PATH, Seattle, WA USA; 5 U. S. President’s Malaria Initiative, USAID, Washington, DC USA; 6 Audere, Seattle, WA USA

**Keywords:** RDT, MaCRA, TPR, Malaria Data Accuracy

## Abstract

**Background:**

Malaria remains a major health concern in Nigeria. Rapid diagnostic tests (RDTs) are widely used in health facilities to confirm malaria before treatment. However, concerns remain about healthcare workers (HCWs) adherence to, and reporting of test results. This study assessed the accuracy of RDT results recorded in health facility registers in two states of Nigeria by comparing them with an unbiased reference standard and explored factors influencing interrater agreement.

**Methods:**

A mixed-method evaluation was conducted in 16 health facilities across Oyo and Sokoto States. RDTs performed by HCWs were photographed using a digital RDT reader and independently re-interpreted by a trained, independent, objective panel. Surveys of health facilities and HCWs collected data on factors that could influence RDT recording. Interrater agreement between RDT results recorded by HCWs in facility registers and the external panel was assessed using Cohen’s kappa. A meta-analytical approach was used to calculate a pooled summary kappa value across facilities, and potential moderators of agreement were examined, including characteristics of facilities, HCWs and RDTs.

**Results:**

Out of 19,586 RDTs captured, 18,319 were included in the analysis. Overall, 6.2% of RDTs were misrecorded as positive and 3.7% as negative in health facility registers, yielding a positive predictive value of 87.2% (95% confidence interval [CI] 86.4%, 87.8%) and negative predictive value of 92.9%. The overall percentage agreement was 90.2% (95% CI 89.7%, 90.6%), and the pooled kappa statistic was 0.80 (95% CI 0.75, 0.85), indicating strong agreement. However, kappa values varied substantially across facilities (range: 0.59, 0.92). Lower agreement was observed in facilities in Sokoto State and in areas with lower malaria prevalence and test positivity. Faint test lines, found in 8.8% of RDTs, were associated with a significantly increased likelihood of results misrecorded as negative. HCWs were more likely to misrecord RDT results as positive when a malaria diagnosis or antimalarial prescription had been made.

**Conclusion:**

While overall agreement between facility registers and panel-interpreted RDT results was strong, the proportion of results misrecorded as positive and negative highlight the need for improved training, supportive supervision, and mechanisms to promote accurate RDT interpretation and recording. Targeted interventions are essential to ensure the reliability of routine malaria data and support national control efforts.

## Background

Malaria is a major public health concern in Nigeria, with an estimated 68 million cases and 184,689 deaths attributed to the disease in 2023, accounting for 26% of the global malaria burden [1]. Malaria is endemic across Nigeria, with transmission rates influenced by ecological, climatic, and socio-economic factors [2]. Despite being treatable and preventable, malaria imposes a substantial burden on the Nigerian healthcare system [2]. The disease disproportionately affects children under 5 years old and pregnant women, contributing to high hospitalization rates, economic losses, and significant mortality [3]. Promisingly, from 2010 to 2021, the prevalence of malaria by microscopy in Nigeria declined significantly from 42 to 22% [4, 5]. This decline is significantly attributed to efforts led by the National Malaria Elimination Programme (NMEP) and other supporting organizations to strengthen malaria prevention, vector control and case management through various interventions.

Case management is a core component of the NMEP’s malaria strategy, and accurate malaria diagnosis is essential for ensuring appropriate treatment and promoting better health outcomes. The World Health Organization (WHO) recommends parasitological confirmation of malaria either through microscopy or malaria rapid diagnostic tests (RDTs) prior to treatment [6]. RDTs have become essential tools in malaria diagnosis, offering a simple, accurate, and inexpensive means of detecting *Plasmodium* antigens in human blood compared to microscopy [7]. Research has shown that RDTs perform almost as accurately as microscopy for detecting malaria [8], require minimal training, provide rapid results, and do not rely on complex equipment [9]. In 2012, Nigeria adopted RDTs as part of its malaria case management strategy to improve the specificity of malaria diagnosis, ensuring that only patients with confirmed malaria receive artemisinin-based combination therapy (ACT) [10]. This approach aimed to reduce the overuse of antimalarial medicines, promote targeted treatment, and enhance malaria case management outcomes.

The expanded use of RDTs has not only strengthened malaria case management in Nigeria but also improved the accuracy of malaria surveillance data collected through health facility records and community health workers [11]. With increased availability of RDTs, rates of parasitological confirmation have increased, improving the specificity of malaria diagnosis. With the adoption of the District Health Information Software 2 (DHIS2) in 2010, confirmed malaria cases can be reported more systematically, enabling Nigerian health authorities to make better-informed, data-driven decisions regarding resource allocation [12].

The test positivity rate (TPR) is a key surveillance indicator recommended by the WHO for use in areas of high malaria transmission. It is calculated as the number of positive tests divided by the total number of tests performed [13]. The use of TPR from health facility records assumes that individuals seeking care for suspected malaria are broadly representative of the general population. However, TPR can be influenced by the incidence of non-malarial febrile illnesses, which may affect care-seeking behaviour and testing patterns and inflate the denominator. Despite this, studies comparing TPR with malaria incidence rates obtained through passive surveillance have shown that TPR serves as a suitable proxy for transmission intensity and is not significantly biased by proximity to health facilities [14].

Although RDTs are widely used in Nigeria, particularly in rural health facilities where microscopy is often unavailable, their accuracy depends on proper administration and interpretation [15]. Concerns remain regarding healthcare workers’ (HCWs’) ability to use RDTs correctly and adhere to their results, especially in cases of negative outcomes [16]. This continues to pose challenges in Nigeria, as adhering to negative RDT outcomes requires refraining from prescribing antimalarials without confirmed malaria infection. In Oyo State, a cross-sectional survey found that 26% of HCWs prescribed antimalarials to patients with negative RDT results [17]. A similar survey in Ebonyi State reported an even higher proportion, with 50.8% of HCWs indicating that they had prescribed antimalarials to patients with a negative test result [18]. Analysis of nationally-representative samples from Nigeria Malaria Indicator Surveys from 2010, 2015 to 2021 showed that 36.1% of children who tested RDT-negative were nonetheless prescribed an antimalarial.

The ratio of antimalarial treatments to laboratory-confirmed malaria cases is frequently used to monitor the rational use of antimalarials and to detect potential leakage in the supply chain [19]. When HCWs administer antimalarials despite negative RDT results, this ratio decreases and may raise red flags. To avoid scrutiny over discrepancies in this indicator, HCWs may be motivated to misrecord a negative RDT result as positive in health facility registers when providing treatment, irrespective of test outcome. This practice inflates the TPR and may obscure meaningful trends in malaria burden across time and space.

RDT results recorded in health facility registers have rarely been systematically and objectively evaluated against the results indicated on the test cassettes [20]. To address this gap, a multi-country study was conducted to assess the interrater agreement between RDT results recorded by HCWs in health facility registers and those of a trusted reference standard. Factors that could moderate the level of interrater agreement were also examined, and the implications for malaria case management and surveillance were considered. This paper reports presents findings from the Nigeria component of the study.

## Methods

Detailed methods for this mixed-methods evaluation can be found in the first paper in this series [21]. Aspects of the methods that were specific to Nigeria are described below.

### Study sites

To ensure uninterrupted availability of RDTs and antimalarials during the study period, two states were selected from among the 11 supported by the US President’s Malaria Initiative (PMI). One state each from Nigeria’s northern and southern regions was purposively selected to capture regional differences in malaria transmission intensity and seasonality. The states with the highest TPR reported on the DHIS2 in each region (Sokoto in the north and Oyo in the south) were chosen. Within each state, two local government areas (LGAs) with high TPR, one urban and one rural, were selected.

The selection of health facilities within the LGAs has been described previously [21]. Health facilities that reported a minimum average of 50 RDTs per month and had reported data on RDT to the DHIS2 for at least nine months out of 12 over the previous 2 years were stratified by median TPR and RDT volume. This was to ensure the inclusion of facilities with adequate RDT testing volume and consistent reporting, in order to meet the study’s sample size requirement and measure impact post-implementation. One health facility was randomly selected from each stratum to participate in the study. However, one facility in Oyo had to be dropped due to inaccessibility and security concerns, and there was no facility within the same stratum for substitution. Therefore, a facility in a different stratum had to be selected, resulting in an unbalanced distribution by stratum. A total of 16 primary healthcare facilities (classified as level 1 facilities in Nigeria) were included in the study.

### Data collection

Data were collected by trained research assistants in the selected health facilities using standardized data collection tools designed in KoboToolbox (Kobo, Cambridge, MA USA) and the HealthPulse application (Audere, Seattle, WA USA).

### Facility survey

A baseline facility survey was conducted to assess the operational characteristics of each selected health facility. The survey included: a count of the HCWs working in the facility by cadre; presence of registers, guidelines and job aids; availability of laboratory diagnostic test kits; first- and second-line antimalarial medicines; stockouts of medicines and supplies over the previous 3 months (a stockout was described as at least seven consecutive days without the medicine or supply); and status of infrastructure such as electricity and internet connection.

### Knowledge, attitudes, perceptions, and behaviour (KAPB) survey

A knowledge, attitudes, perceptions and behaviour (KAPB) survey was administered to HCWs engaged in RDT testing, interpretation, treatment or record-keeping to collect detailed information on their training, experience, knowledge, attitudes, perceptions, behaviours, and subjective norms concerning malaria RDTs. Unique identifiers were generated for HCWs who participated in the survey and were used with the HealthPulse app to link data to HCWs characteristics. Research assistants observed HCWs performing an RDT and utilized a 19-point standardized checklist to assess their proficiency.

### RDT interpretations

Research assistants used the HealthPulse app to capture images of RDTs as soon as possible after they were interpreted by HCWs without interfering with patient care. Unique identifiers for the HCW who performed the RDT and the HCW who entered the RDT data into the health facility register were recorded separately. The age and sex of the patient on whom the RDT was performed was recorded from the health facility register along with the RDT result, diagnosis and prescribed treatment.

RDT images were sent to a trained and quality-controlled external panel who interpreted the RDT outcome from the photo according to the manufacturer’s instructions as positive, negative or invalid. The panel noted the type of RDT product, which included Bioline Malaria Ag P.f. (Abbott, IL USA), AdvDx Malaria Pf Rapid Malaria Ag Detection Test (Advy Chemical, Mumbai, India), CareStart Malaria Pf Ag (Access Bio Inc, New Jersey, USA) and First Response Malaria Antigen *P. falciparum* (HRP2) Card Test (Premier Medical Corporation Ltd, Gujarat, India). The panel noted faint test lines and also documented any anomalies detected with the RDT, including blood in the result window and blood obscuring result lines.

### Data management and analysis

Data were analysed using R (R Foundation for Statistical Computing, Vienna, Austria). The geolocation of each facility was used to derive the average parasite prevalence for 5 km around each facility using the malariaAtlas package in R [22]. HCWs were grouped into terciles based on their RDT proficiency index comprising aspects related to biosafety, testing procedures and the accuracy of the RDT interpretation. The percentage agreement between RDT results recorded in health facility registers and those determined by the external panel was calculated, along with the positive and negative predictive values (PPV and NPV) of register-recorded RDT results. Ninety-five percent confidence intervals for these metrics were computed using Wilson binomial methods. Agreement between HCWs and the external panel on RDT results was assessed using Cohen’s kappa statistic, which adjusts for the possibility of chance agreement and is conceptually similar to a correlation coefficient [23]. A custom R function was developed to summarize kappa values across health facilities by calculating a weighted mean, with weights based on the inverse of the variance for each facility [24]. In addition to estimating a pooled kappa statistic, potential sources of variation in agreement were explored by assessing key characteristics of health facilities, HCWs and RDTs as moderators.

### Ethical considerations

The study received ethical review and approval by the Nigeria National Health Research Ethics Committee and the state research ethics review committees of Oyo and Sokoto states. The WIRB-Copernicus Group (WCG) institutional review board (Cary, NC) also reviewed and approved the protocol.

Prior to data collection, written, informed consent was obtained from all HCWs who participated in the study. No written consent was obtained from patients as secondary data sources (i.e., patient registers) were used to record patients’ encounter anonymously. Cconfidentiality was maintained by providing training to data collectors, restricting data access through encrypted servers, and ensuring anonymity of collected data. The database and consent forms from the study will be destroyed 3 years after the study ends.

## Results

**Fig. 1:**
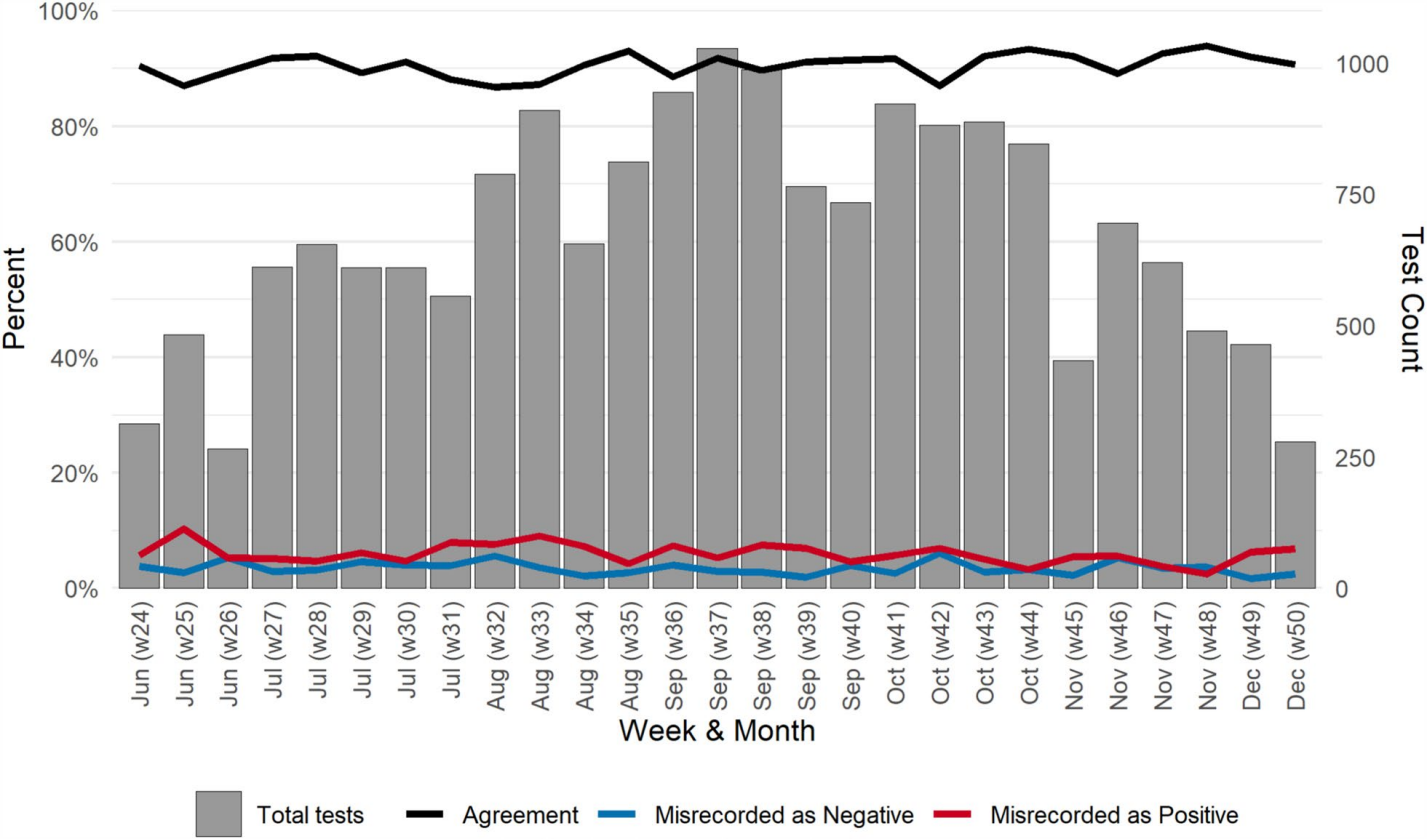
Number of rapid diagnostic tests observed, proportion of results in agreement and proportion misrecorded as positive or negative by week, Nigeria 2023 (n = 18,319). Health facilities 1–8 were in Oyo State and 9–16 were in Sokoto State (The red shaded area indicates the pooled kappa estimate across all health facilities)

A total of 19,586 RDT images with matching patient data were captured from June to December 2023. The final analytical dataset comprised 18,319 RDT observations (93.5%), following the exclusion of 1267 observations due to image quality problems, absence of RDT result in the health facility register, and missing healthcare worker codes. The number of RDTs per week rose after the start of the study in June, peaked in the middle of the study in September with 3912 observations, and then declined towards the end of the study in mid-December (Fig. 1).

### Interrater agreement on rapid diagnostic tests results

There were 8792 (48.0%) positive results recorded in the health facility register and 9527 (52.0%) negative results. In contrast, the external panel classified 8302 (45.3%) RDTs as positive, 9953 (54.3%) as negative, and 64 (0.3%) as invalid. HCWs and external panel were in agreement for 7663 (41.8%) positives and 8852 (48.3%) negatives. Although the panel identified 64 RDTs as invalid, no RDTs were recorded as invalid in the health facility register. There were 1101 (6.2%) RDTs misrecorded as positive in the health facility register and 639 (3.7%) misrecorded as negative, leading to a PPV of 87.2% (95% CI, 86.4%, 87.8%) and a NPV of 92.9% (95% CI 92.4%, 93.4%).

The percentage agreement between HCWs and the external panel was 90.2% (95% CI 89.7%, 90.6%). Percentage agreement varied slightly week-to-week during the study period with a narrow interquartile range (IQR) of 88.9–91.6% (Fig. 1). The proportion of results misrecorded as positive (IQR 4.9–7.1) was higher than the proportion misrecorded as negative (IQR 2.7–4.0) for all but 2 weeks of the study.

**Fig. 2:**
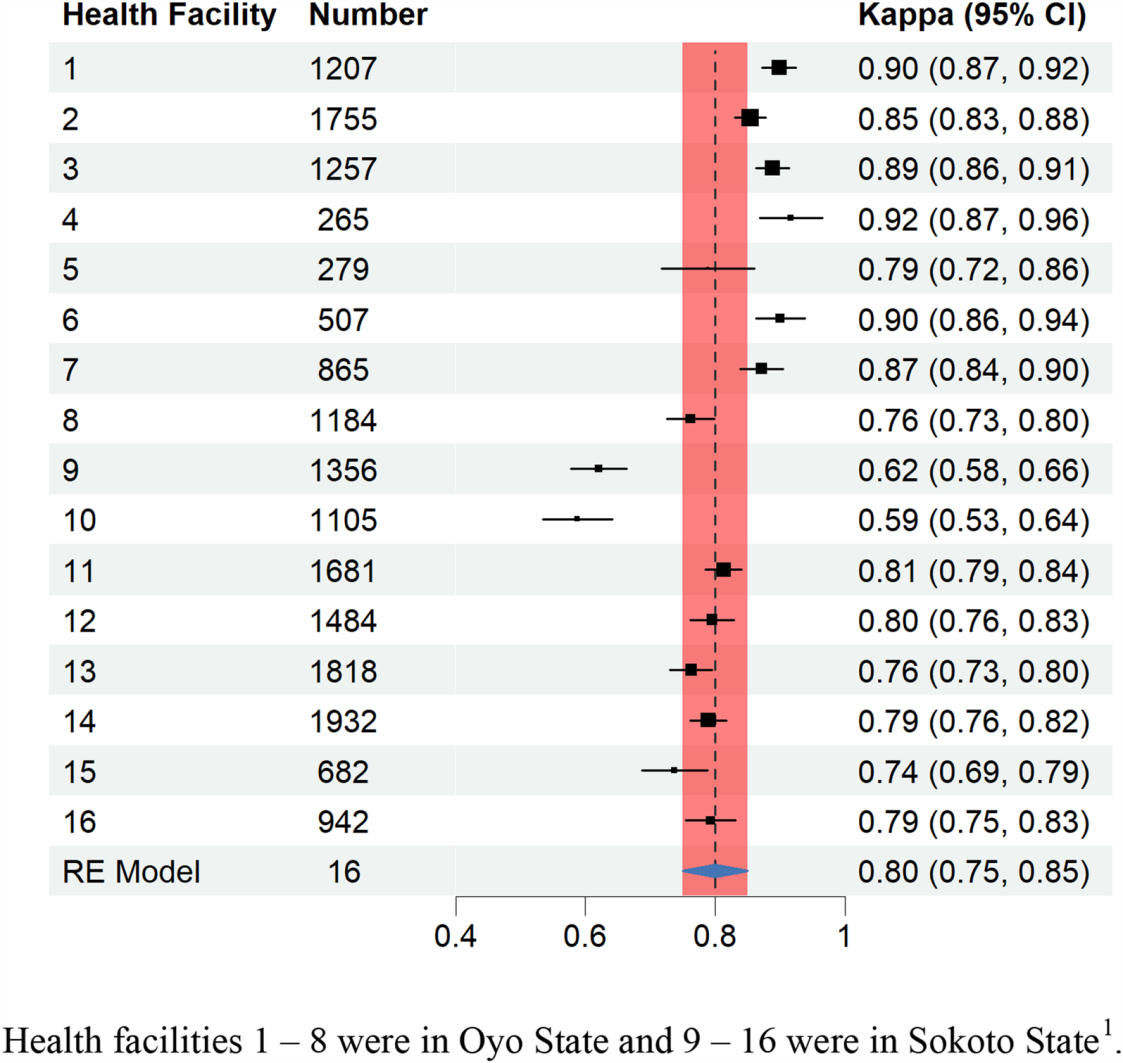
Forest plot of agreement on rapid diagnostic test results between healthcare workers and an external panel in 16 health facilities, Nigeria 2023

The summary value for interrater agreement from the meta-analytical random effects model was 0.80 (95% confidence interval [CI] 0.75, 0.85), indicating strong agreement between RDT results recorded in health facility registers and the external panel reviewing RDT images (Fig. 2). Kappa scores for individual health facilities ranged from 0.59 (95% CI 0.53, 0.64) to 0.92 (95% CI 087, 0.96). Six health facilities, all located in Oyo State, had higher kappa scores than the summary value with three (health facilities 1, 4 and 6) reaching near-perfect agreement. Two health facilities (9 and 10), both located in Sokoto State, had much lower scores ranging from weak to moderate levels of agreement (Fig. 2).

### Characteristics of health facilities

Among the 16 facilities selected for this study, most (14, 87.5%) were located in areas where malaria prevalence ranged between 10 and 29% (Table 1). A plurality of facilities (7, 43.8%) had at least five staff performing RDTs. Laboratory technicians were present in 11 facilities (68.8%), and case management guidelines and job aids for RDTs were available in 14 facilities (87.5%).

**Table 1:** Key characteristics of health facilities, numbers of rapid diagnostic tests (RDTs) recorded, interrater agreement and proportion of RDT results that were misrecorded, Nigeria 2023

Characteristic	Health facilities N = 16 n (%)	RDTs N = 18,319 n (%)	Cohen’s kappa (95% CI)	Misrecorded as:	
				Positive %	Negative %
State					
Oyo	8 (50.0)	7319 (40.0)	0.86 (0.81, 0.91)	4.5	2.5
Sokoto	8 (50.0)	11,000 (60.0)	0.74 (0.69, 0.79)	7.3	4.5
Local government area					
Ibadan SW, Oyo State	4 (25.0)	4484 (24.5)	0.89 (0.82, 0.96)	3.5	2.3
Oluyole, Oyo State	4 (25.0)	2835 (15.5)	0.83 (0.76, 0.90)	6.0	2.9
Wamako, Sokoto State	4 (25.0)	5626 (30.7)	0.71 (0.64, 0.78)	7.6	5.2
Wurno, Sokoto State	4 (25.0)	5374 (29.3)	0.77 (0.70, 0.84)	7.0	3.7
Stratum					
High volume/High TPR	4 (25.0)	5488 (30.0)	0.84 (0.74, 0.93)	5.4	2.6
High volume/Low TPR	5 (31.2)	6118 (33.4)	0.78 (0.69, 0.86)	6.9	4.1
Low volume/High TPR	4 (25.0)	3167 (17.3)	0.83 (0.73, 0.92)	5.4	3.9
Low volume/Low TPR	3 (18.8)	3546 (19.4)	0.75 (0.64, 0.86)	6.8	4.5
Parasite prevalence (PfPr_2–10_)					
0–9%	0 (0)	0 (0)			
10–19%	7 (43.8)	9319 (50.9)	0.73 (0.68, 0.78)	7.9	4.3
20–29%	7 (43.8)	7537 (41.1)	0.88 (0.83, 0.92)	3.3	3.3
30–39%	2 (12.5)	1463 (8.0)	0.77 (0.68, 0.86)	9.8	2.0
Number of staff who perform RDTs					
1–2	3 (18.8)	3139 (17.1)	0.83 (0.74, 0.93)	7.7	2.2
3–4	6 (37.5)	7652 (41.8)	0.74 (0.67, 0.80)	7.0	4.7
5 or more	7 (43.8)	7528 (41.1)	0.84 (0.78, 0.90)	4.7	3.3
Laboratory technician present					
Yes	11 (68.8)	14,329 (78.2)	0.78 (0.73, 0.83)	6.1	4.2
No	5 (31.2)	3990 (21.8)	0.85 (0.76, 0.93)	6.4	2.0
Case management guidelines present					
Yes	14 (87.5)	14,746 (80.5)	0.80 (0.75, 0.85)	6.3	3.8
No	2 (12.5)	3573 (19.5)	0.81 (0.68, 0.94)	5.4	3.3
Laboratory register present					
Yes	8 (50.0)	10,906 (59.5)	0.77 (0.71, 0.83)	6.7	4.1
No	8 (50.0)	7413 (40.5)	0.83 (0.77, 0.89)	5.3	3.0
RDT job aid present					
Yes	14 (87.5)	16,030 (87.5)	0.82 (0.78, 0.86)	5.5	3.5
No	2 (12.5)	2289 (12.5)	0.68 (0.56, 0.79)	9.7	4.8

*CI* confidence interval; PPfPr_2–10_: *P. falciparum* parasite prevalence in children 2–10 years old; *RDT* rapid diagnostic test; *TPR* test positivity rate

More RDTs were recorded in Sokoto (11,000, 60.0%) than in Oyo State (7319, 40.0%) (Table 1). In particular, Wamako LGA (Sokoto State) was responsible for the largest number of RDTs recorded (5625, 30.7%) among the LGAs. As expected, the health facilities that were selected from the two high patient volume strata recorded more RDTs than those from the two low volume strata. Health facilities with a laboratory technician (11, 68.8%) contributed a greater proportion of RDTs (14,329; 78.2%) than would be expected given their proportion among all health facilities. The distribution of RDTs by the remaining health facility characteristics generally reflected the distribution of health facilities.

Few health facility characteristics were found to moderate the level of interrater agreement (Table 1). The two LGAs in Sokoto State showed lower agreement compared to the two LGAs in Oyo State, although the confidence intervals overlapped. In both Sokoto LGAs, the proportions of results misrecorded as either positive or negative were higher than those observed in Oyo State. Similarly, while the two strata with lower TPRs exhibited lower levels of agreement, their confidence intervals also overlapped with those of the other strata. Notably, health facilities located in areas with 20–29% parasite prevalence had higher agreement than those in areas with lower or higher prevalence. In these settings of moderate malaria burden, the overall proportion of results that were misrecorded was lower than in settings with lower or higher malaria burden.

### Characteristics of healthcare workers

There were 137 HCWs in study facilities engaged in performing RDTs or recording results of whom 134 (97.8%) were interviewed. When the study concluded, 114 HCWs were found to have contributed at least one RDT recording to the analytical data set. The majority (86, 75.4%) of HCWs were female and aged less than 39 years old (89, 78.1%) (Table 2). Few of the HCWs engaged in recording RDT results were nurses (11, 9.6%), compared with community health workers (38, 33.3%), lab technicians (28, 24.6%) and nonmedical or volunteer staff (27, 23.7%). All HCWs had at least a secondary school education, and almost one quarter (27, 23.7%) were in their first year of work experience. The majority (96, 84.2%) performed RDTs every day.

**Table 2:** Key characteristics of healthcare workers, numbers of rapid diagnostic tests (RDTs) recorded, interrater agreement and proportion of RDT results that were misrecorded, Nigeria 2023

Characteristic	Healthcare workers N = 114 n (%)	RDTs N = 18,319 n (%)	Kappa (95% CI)	Misrecorded as:	
				Positive %	Negative %
Sex					
Female	86 (75.4)	9983 (54.5)	0.81 (0.75, 0.87)	4.9	3.5
Male	28 (24.6)	8336 (45.5)	0.76 (0.69, 0.83)	7.7	3.9
Age (years)					
< 30	54 (47.4)	9139 (49.9)	0.77 (0.70, 0.83)	6.3	3.4
30–39	35 (30.7)	6825 (37.3)	0.81 (0.75, 0.87)	6.4	4.0
40–49	17 (14.9)	1381 (7.5)	0.83 (0.74, 0.91)	5.4	3.5
50–59	8 (7.0)	974 (5.3)	0.85 (0.74, 0.96)	4.8	3.8
Occupational cadre					
Nurse	11 (9.6)	1871 (10.2)	0.81 (0.71, 0.91)	3.8	4.2
Community health worker	38 (33.3)	4467 (24.4)	0.81 (0.74, 0.87)	6.0	2.8
Lab technician	28 (24.6)	7936 (43.3)	0.78 (0.71, 0.85)	6.4	4.1
Nonmedical or volunteer staff	27 (23.7)	2994 (16.3)	0.74 (0.66, 0.82)	8.5	3.5
Medical auxiliary staff	10 (8.8)	1051 (5.7)	0.86 (0.77, 0.96)	3.1	4.4
Highest educational qualification					
Secondary	28 (24.6)	2172 (11.9)	0.82 (0.74, 0.90)	5.4	2.9
University	86 (75.4)	16,147 (88.1)	0.80 (0.75, 0.85)	6.3	3.8
Years of experience					
0–1	27 (23.7)	3404 (18.6)	0.78 (0.71, 0.86)	4.8	3.5
2–4	25 (21.9)	3980 (21.7)	0.78 (0.71, 0.85)	6.7	3.3
5–9	26 (22.8)	5706 (31.1)	0.81 (0.76, 0.87)	6.5	2.9
10 or more	36 (31.6)	5229 (28.5)	0.80 (0.75, 0.85)	6.2	4.9
Frequency of performing RDTs					
Very often (every day)	96 (84.2)	16,037 (87.5)	0.78 (0.72, 0.84)	6.1	3.8
Once in a while to often	18 (15.8)	2282 (12.5)	0.82 (0.73, 0.92)	6.4	2.6
RDT proficiency score tercile					
Low	35 (34.0)	5512 (31.3)	0.81 (0.74, 0.88)	6.6	2.9
Middle	34 (33.0)	6549 (37.2)	0.77 (0.70, 0.84)	6.2	4.2
High	34 (33.0)	5554 (31.5)	0.79 (0.72, 0.85)	5.7	3.8
Received training on RDTs					
Yes	100 (87.7)	16,823 (91.8)	0.80 (0.75, 0.85)	5.8	3.4
No	14 (12.3)	1496 (8.2)	0.73 (0.66, 0.81)	8.7	4.6

*CI* confidence interval; *RDTs* rapid diagnostic tests

Although female HCWs represented the majority of all HCWs recording RDT results, they accounted for just over half of the observed RDTs (9983, 54.5%) (Table 2). Notable disparities were also observed among other subgroups: laboratory technicians made up only a quarter of HCWs but recorded nearly half of all RDTs (7936, 43.3%), while HCWs with university qualifications recorded the vast majority of RDTs (16,147, 88.1%) despite comprising only three-quarters of HCWs.

None of the HCW characteristics were found to meaningfully moderate the level of agreement between RDT results recorded in health facility registers and the external panel (Table 2). The highest proportion of results misrecorded as positive were attributed to nonmedical or volunteer staff, while the lowest was among medical auxiliary staff.

### Characteristics of the RDT

Four RDT products were used during the study period. The majority of tests were performed using AdvDx Malaria Pf (14,501, 79.2%), followed by First Response Malaria Pf (3645, 19.9%) (Table 3). A small number of tests were conducted using Bioline Pf (142, 0.8%) and CareStart Pf (31, 0.2%).

**Table 3:** Key characteristics of rapid diagnostic tests (RDTs), interrater agreement and proportion of RDT results that were misrecorded, Nigeria 2023

Characteristic	N (%)	Kappa (95% CI)	Misrecorded as:	
			Positive %	Negative %
RDT product				
AdvDx Pf	14,501 (79.2)	0.80 (0.75, 0.84)	6.5	3.4
First Response Pf	3645 (19.9)	0.80 (0.73, 0.86)	4.6	4.8
Bioline Pf	142 (0.8)	0.67 (0.51, 0.84)	13.4	3.5
CareStart Pf	31 (0.2)	0.74 (0.45, 1.00)	12.9	0
Month RDT was performed				
June	1063 (5.8)	0.79 (0.71, 0.87)	7.7	3.7
July	2714 (14.8)	0.76 (0.70, 0.83)	5.7	3.7
August	3391 (18.5)	0.78 (0.72, 0.84)	7.2	3.7
September	3912 (21.4)	0.82 (0.76, 0.88)	6.7	3.0
October	3877 (21.2)	0.79 (0.73, 0.85)	5.9	4.4
November	2559 (14.0)	0.83 (0.77, 0.89)	4.1	3.9
December	803 (4.4)	0.79 (0.71, 0.87)	6.5	2.5
Faint line				
Yes	1619 (8.8)	0.16 (0.06, 0.26)	0.6	23.3
No	16,700 (91.2)	0.83 (0.78, 0.88)	6.7	1.8
Patient sex				
Female	10,928 (59.7)	0.79 (0.74, 0.84)	6.4	3.8
Male	7377 (40.3)	0.81 (0.77, 0.86)	5.8	3.6
Patient age (years)				
0–4	5068 (27.7)	0.80 (0.75, 0.85)	6.3	3.6
5–14	3796 (20.7)	0.81 (0.76, 0.86)	5.7	3.1
15 and more	9455 (51.6)	0.78 (0.73, 0.83)	6.3	4.0
Patient diagnosed with malaria				
Yes	12,165 (66.4)	0.70 (0.64, 0.76)	9.0	2.1
No	6154 (33.6)	0.14 (0.04, 0.25)	0.5	6.7
Patient prescribed antimalarial				
Yes	9526 (52.0)	0.41 (0.29, 0.52)	11.1	1.0
No	8793 (48.0)	0.26 (0.13, 0.39)	0.8	6.6
RDT performed and recorded by the same person				
Yes	17,896 (97.7)	0.80 (0.76, 0.85)	6.1	3.6
No	423 (2.3)	0.61 (0.50, 0.72)	8.7	6.1

*CI* confidence interval; *RDT* rapid diagnostic test

Most RDTs were performed between September (3912, 21.4%) and October (3877, 21.2%) (Table 3). A substantial proportion of the RDTs were noted to have faint lines (1619, 8.8%). Among the patients on whom RDTs were performed, 10,928 (59.7%) were female and the majority were aged 15 years or above (9455, 51.6%). Malaria was diagnosed in 12,165 cases (66.4%) and ACTs were prescribed in 9526 (52.0%) instances. Nearly all RDTs were performed and recorded by the same person (17,896, 97.7%).

Interrater agreement was moderated by several characteristics of RDTs and the patients for whom they were performed (Table 3). The level of agreement was 0.16 (95% CI 0.06, 0.26) when an RDT had a faint line compared to 0.83 (95% CI 0.78, 0.88) without a faint line. RDTs with faint lines were much more likely to be misrecorded as negative (23.3%) than misrecorded as positive (0.6%). The interrater agreement was also modified by a diagnosis of malaria, prescription of antimalarials and RDTs performed and recorded by different people. The proportion of results misrecorded as positive was much higher when a patient was diagnosed with malaria (9.0% vs. 0.5%) while the proportion of results misrecorded as negative was much lower when they were diagnosed with malaria (2.1% vs. 6.7%). A similar trend was found with prescription of antimalarial medicines. RDT results not performed and recorded by the same person were associated with higher proportions of both results misrecorded as positive (8.7%) and negative (6.1%).

## Discussion

This study demonstrated a strong level of agreement between RDT results recorded in health facility registers and results from an objective reference standard across two states in Nigeria. Despite this encouraging finding, notable discrepancies remain: 6.2% of tests overall were misrecorded as positive and 3.7% as negative. These misclassifications suggest there is substantial room for improvements in the accuracy of RDT results reported to the national health management information system. Furthermore, the positive predictive value of 87.4% indicates that one in nearly nine positive RDT results recorded in health facility registers may not represent a true malaria case, underscoring the need for additional interventions to improve data accuracy.

Health facilities demonstrated substantial variability in interrater agreement on RDT results, ranging from near-perfect agreement in some facilities to markedly lower levels in others. Interrater agreement was lower in Sokoto State compared to Oyo State, suggesting potential interstate variation that may reflect differences in training quality and coverage, availability of resources, or systematic differences in malaria reporting practices. The higher level of agreement observed in Oyo State may partly be attributed to prior contextual factors; notably, the RDT validation exercises that were already ongoing in Oyo prior to the MaCRA assessment [20]. These earlier activities could have enhanced provider familiarity and proficiency with RDT use and interpretation, and motivated improved consistency in test results.

Agreement was also lower in facilities with lower test positivity rates and lower parasite prevalence. In these settings, both misrecording of positives and negatives was higher, suggesting that a lower malaria burden may be associated with reduced familiarity or confidence in interpreting RDT results. This corroborates a study conducted in Zambia [25]. Furthermore, the significant variability in interrater agreement across health facilities suggests that factors beyond test interpretation such as differences in training, supervision, workload, and facility infrastructure may influence recording accuracy [15, 21]. Facilities with near-perfect agreement (e.g., three in Oyo State) may likely have benefited from structured training programs and regular supervision, whereas those with lower agreement (e.g., two in Sokoto State) may lack these supports. Findings from a previous study indicate that targeted training and consistent supervision are critical for effective malaria RDT reporting [26].

Several characteristics of RDTs were notably associated with interrater agreement. In particular, faint lines were associated with a much higher likelihood of results being misrecorded as negative. Although faint lines were observed in only 8.8% of all RDTs in the study, this seemingly small proportion could have significant implications if generalizable across Nigeria. With over 29 million RDTs performed annually, this could translate to over half a million true positive cases being misrecorded as negative, potentially resulting in missed opportunities for appropriate treatment [1]. There are several possible reasons why HCWs may miss faint lines, including interpreting results too early, lighting conditions, hospital infrastructure or inadequate attention to test results. Given the potential scale of this issue, health officials should reinforce adherence to RDT instructions, emphasize the importance of careful interpretation of negative results and consider routine assessment of HCW visual acuity. In addition, regular refresher training has been recommended to reduce misdiagnosis [27]. The use of digital tools and artificial intelligence as advocated in [21, 28] is highly recommended to reduce the subjectivity in faint lines interpretation. However, cost and technical know-how might create a barrier.

Patient diagnosis and type of treatment were strong moderators of interrater agreement. Individuals with a recorded malaria diagnosis or an antimalarial prescription were much more likely to have RDT results misrecorded as positive and much less likely to have results misrecorded as negative compared to the overall pattern. These findings suggest that some HCWs may misrecord RDT results to align with clinical decisions they have already made. While laboratory diagnostics are intended to support clinical decision-making, pressure to adhere strictly to test outcomes may lead HCWs to record results that justify treatment decisions rather than reflect the actual test outcome. Health authorities should consider strategies that encourage accurate recording of RDT results while also promoting adherence to test results.

### Strengths and limitations

A key strength of this study was the use of an external panel trained to interpret RDT images as the reference standard. This approach offered a standardized, unbiased, and quality-controlled method for evaluating RDT results and enabled the review of over 18,000 tests. However, the use of photographs rather than physical RDTs introduces a potential limitation. Evidence suggests that faint test lines may be more easily identified with the naked eye than in digital images [29], which could affect the accuracy of the panel’s classifications. While RDT results were considered to be accurately recorded when they matched the external panel’s interpretation, the panel itself does not represent a perfect gold standard. As such, some degree of misclassification in interrater agreement is possible. Nonetheless, given the large sample size, it is unlikely that this would have meaningfully altered the study’s main findings. This study is also limited in its generalizability given that the assessment was conducted in only 16 health facilities across two states. Additionally, the period covered by the study does not reflect the full seasonal variation of malaria transmission in Nigeria.

## Conclusions

This study provides important insights into the accuracy of routine malaria RDT reporting in Nigerian health facilities. While overall agreement between facility registers and external panel interpretations was high, the proportion of results misrecorded as positive and negative underscore the need for targeted interventions to enhance the reliability of routine data and support effective malaria control efforts. Innovative strategies such as monthly validation of health facility register data against archived RDT cassettes, which has already been implemented in some states in Nigeria, should be explored to improve data quality throughout the country.

### Further studies

This research has opened areas that could warrant further research exploration. Hence, while this study provides robust evidence of factors affecting accurate reporting of malaria RDT in low resource settings, several questions remained unanswered:

- Would results differ in non-PMI-supported states? Facilities with less consistent RDT supplies may show higher misclassification.
- Would results differ across the six geopolitical regions of Nigeria? This study reported results from two out of the six regions of the country. Hence future studies could compare results across the regions.
- How does seasonality affect accuracy? Malaria peaks might increase workload and odds of errors. Future studies could compare results in rainy and dry (harmattan) seasons.
- What is the clinical impact of misrecorded results? Future studies could track patient outcomes based on recording accuracy.

## Data Availability

The datasets used and/or analysed during the current study can be provided by the corresponding author on reasonable request.

## Abbreviations

ACT: Artemisinin-based Combination Therapy
BMGF: Bill and Melinda Gates Foundation
DHIS2: District Health Information Software 2
HCFs: Healthcare Facilities
HCWs: Healthcare Workers
LGAs: Local Government Areas
RDT: Rapid Diagnostic Test
NMEP: National Malaria Elimination Programme
PMI: President’s Malaria Initiative
PfPR: *Plasmodium falciparum* Parasite prevalence
GPR: Geopolitical Regions
RDT: Rapid Diagnostic Test
SSA: Sub-Saharan Africa
TPR: Test Positivity Rate
WHO: World Health Organization
